# 

**Published:** 1853-10

**Authors:** 


					THE
AMERICAN JOURNAL
DENTAL SCIENCE.
EDITED BY
CHAPIN A. HARRIS, M. D., D. D. S.
ALFRED A. BLANDY, M. D., D. D. S.
A. SNOWDEN PIGGOT, M. D.
NEW SERIES?VOLUME IV.
PHILADELPHIA:
LINDSAY & BLAKISTON.
BALTIMORE:?ARMSTRONG & BERRY.
LONDON:?TRUBNER & CO., 12 Paternoster Row.
1853
w
1
AM
4-6
M
JOHN TV . WOODS, PRINTER,
BALTIMORE.

				

